# Gas Under Diaphragm: A Rare Case of Ruptured Liver Abscess With Gas Forming Organism

**DOI:** 10.7759/cureus.21672

**Published:** 2022-01-27

**Authors:** Abdul Majeed Maliyakkal, Vamanjore A Naushad, Omran I Al Mokdad, Fathima Hanana, Sahiba M Basheer, Jafer Ajanur Palaki

**Affiliations:** 1 Department of Medicine, Hamad Medical Corporation, Doha, QAT; 2 Medicine, Weill Cornell Medicine-Qatar, Doha, QAT; 3 Medicine, College of Medicine, QU Health, Qatar University, Doha, QAT; 4 Department of Radiology, Hamad Medical Corporation, Doha, QAT; 5 Internal Medicine, Kasturba Medical College, Manipal, IND; 6 Cardiology, Malabar Institute of Medical Sciences, Calicut, IND

**Keywords:** gas under diaphragm, percutaneous drainage, gas forming organism, klebsiella liver abscess, pyogenic liver abscess

## Abstract

Acute abdominal pain with free air under the diaphragm visible on chest/abdomen X-ray (pneumoperitoneum) is a medical emergency. Most of such cases of pneumoperitoneum are attributable to perforated hollow viscus; however, other possibilities like rupture of liver abscess (by a gas-forming organism) also need to be considered. Further imaging like a CT scan might help in the diagnosis and would also obviate the need for laparotomy in some of such cases. We report a case of acute abdominal pain with gas under the diaphragm due to a ruptured liver abscess caused by *Klebsiella pneumoniae*. The patient was managed successfully with ultrasound-guided percutaneous aspiration and insertion of a drain along with antibiotics and supportive measures, and no laparotomy was performed.

## Introduction

Acute abdominal pain with gas under the diaphragm is a medical emergency, and the most common cause is the perforation of a hollow viscus that constitutes about 90% of the cases [1]. Other causes include intra-abdominal sepsis by gas-forming organisms, vaginal insufflation for tubal patency test, overlying bowel gas (Chilaiditi syndrome), entero-biliary fistula, post laparotomy/laparoscopic procedure, gall stone ileus, retroperitoneal air, incompetent sphincter of Oddi, focal biliary lipomatosis, post scuba diving, and pneumatosis coli [2,3]. Ruptured liver abscess by a gas-forming organism is an infrequent cause of gas under the diaphragm. We report a case of gas under the diaphragm due to a ruptured liver abscess caused by *Klebsiella pneumoniae.*

## Case presentation

A 58-year-old gentleman of southeast Asian origin was admitted with complaints of generalized tiredness and fatigability for two weeks, and epigastric and right shoulder pain for the one-week duration. He denied fever, change in bowel habits, or respiratory complaints. His past medical history includes hypertension and Type 2 diabetes mellitus. Vital signs on admission were temperature: 36.7°C, pulse rate: 112/minute, blood pressure (BP): 123/71 mmHg, respiratory rate: 18/ minute, SpO_2_: 99% breathing on room air. Abdominal examination revealed tenderness over the epigastric area and right hypochondrium. There was no evidence of free fluid in the abdomen. A review of other systems was unremarkable.

Initial investigations showed neutrophilic leukocytosis with elevated inflammatory markers. The metabolic profile revealed elevated blood urea with normal creatinine, hyponatremia, and deranged liver function test. Random blood glucose showed severe hyperglycemia (43.1 mmol/L) and raised serum beta-hydroxybutyrate (1.6 mmol/L). A blood gas study showed a normal pH and bicarbonate. Serum lactate level was mildly elevated (Table 1).

**Table 1:** Serial laboratory results Hgb: Hemoglobin. WBC: White blood cell. CRP: C-reactive protein. ALT: Alanine aminotransferase. AST: Aspartate aminotransferase. ALP: Alkaline phosphatase.

Tests (normal range)	On admission	7^th^ day of admission	5 weeks (on the day of discharge)	1 month after discharge
WBC (4-10 × 10^9^/L)	14.9	16	8.4	8.1
Neutrophils (2-7 × 10^9^/L)	14.3	13.3	5.7	4.1
Hgb (12-15 gm/dL)	11.9	8.4	10.2	11
Platelet (150-400 × 10^3 ^/ µL)	185	523	405	327
CRP (mg/L)	289	126	11.2	<2
Procalcitonin(ng/mL)	16.10	0.79	-	-
Serum sodium (133-146 mmol/L)	120	136	135	146
Potassium (3.5-5.3 mmol/L)	3.7	3.6	4.4	4.8
Chloride (95-108 mmol/L)	86.7	123	96	99
Bicarbonate (22-29 mmol/L)	22.1L	21	29	24
Blood urea (2.5-7.8 mmol/L)	16.9	2.7	6.8	4.8
Serum Creatinine (62-106 umol/L)	94	57	71	68
Random Glucose (3.3-5.5 mmol/L)	43.1	5.9	7.2	-
Serum lipase (8-78 U/L)	99	-	-	-
Lactate (0.5-2.2 mmol/L)	2.4	1.4	-	-
Total bilirubin (3.5-24 µmol/L)	9.1	12	4	6
AST (0-30 U/L)	601	47	34	15
ALT (0-31 U/L)	547	71	55	20
ALP (45-129 U/L)	650	588	135	117
HbA1c (%)	-	14.9	-	-
pH arterial (7.35-7.45)	7.37	-	-	-
Beta hydroxybutyrate (0.0-0.6 mmol/L)	1.6	-	-	-

**Figure 1:**
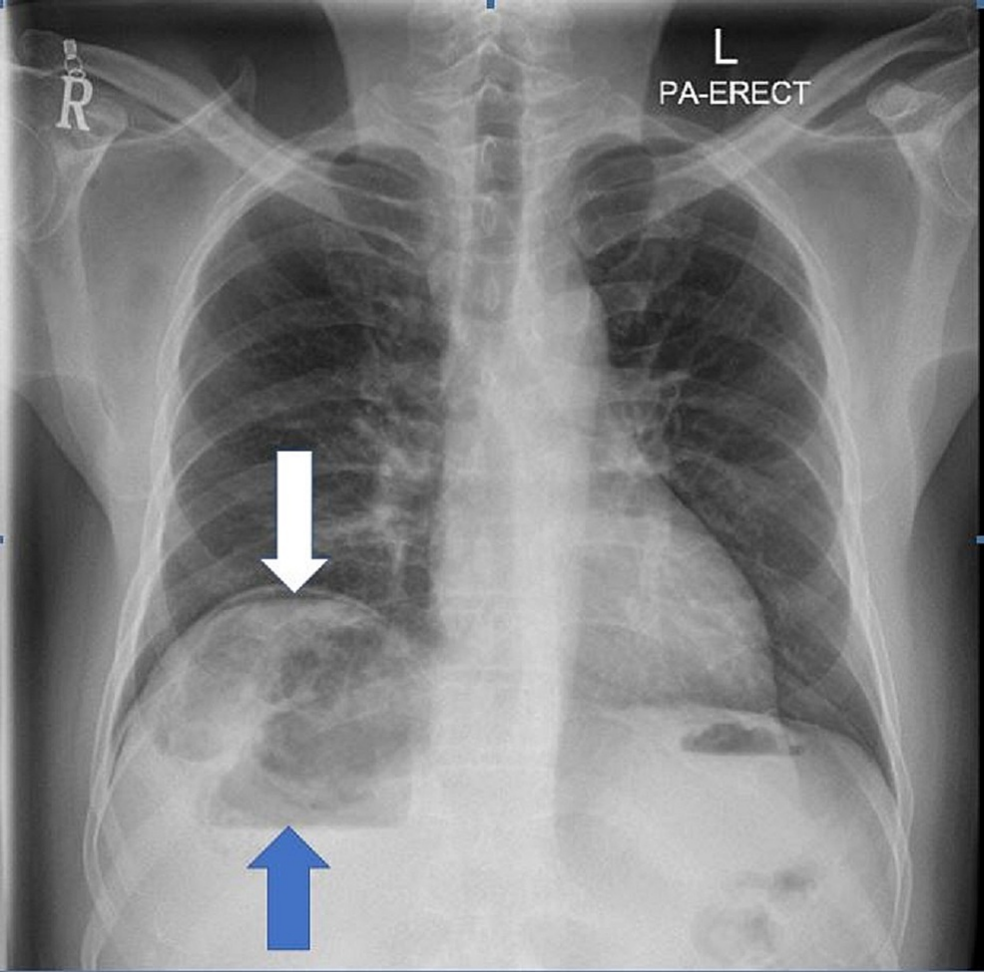
Chest X-ray erect (postero-anterior view) The white arrow is showing right sub-diaphragmatic free air suggesting pneumoperitoneum, with heterogeneous liver shadow and air-fluid level shown by the blue arrow

Chest X-ray revealed right subdiaphragmatic free air suggesting pneumoperitoneum, with heterogeneous liver shadow and air-fluid level (Figure 1).

**Figure 2:**
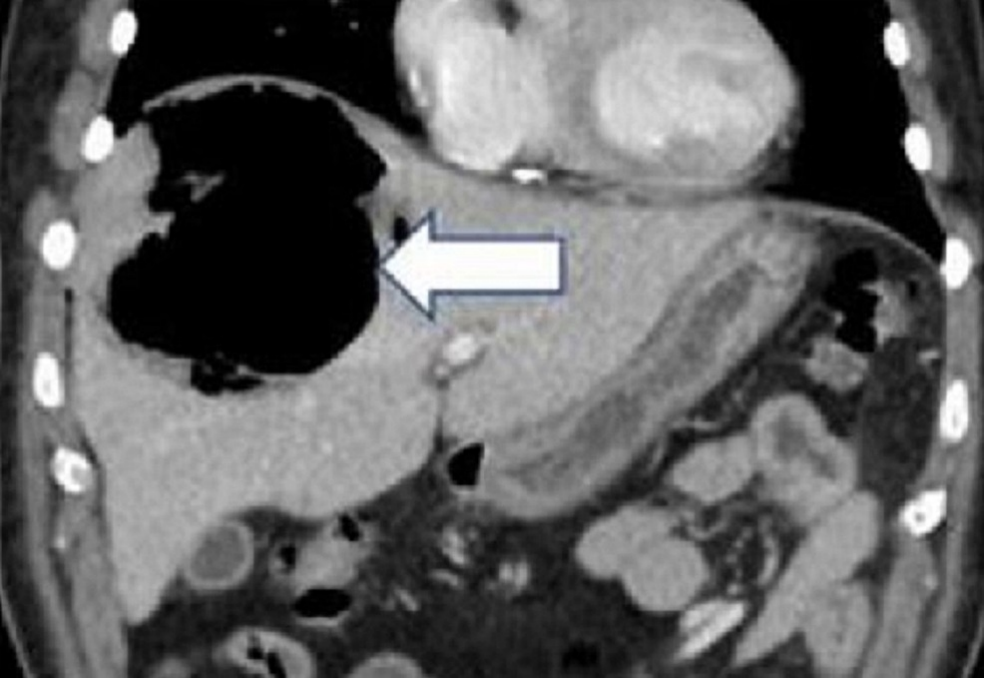
CT scan of the upper abdomen with IV contrast (coronal view) IV: Intravenous The white arrow shows a large cavity like liver abscess mainly containing gas

**Figure 3:**
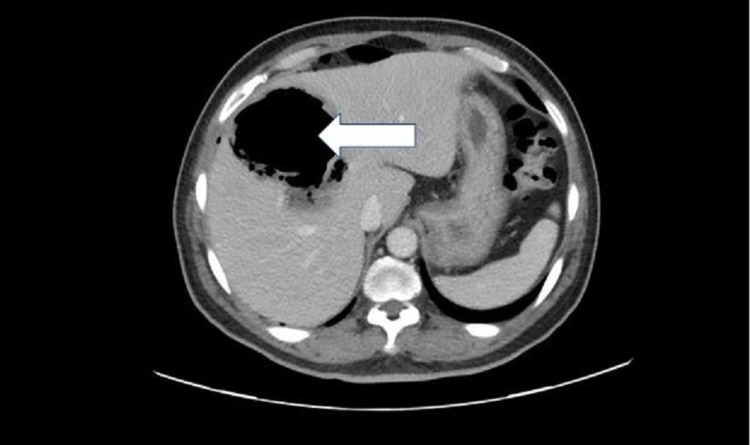
CT scan of the abdomen with IV contrast (axial view) IV: Intravenous The white arrow is showing  gas-containing liver abscess appearing as a hypodense cavity

**Figure 4:**
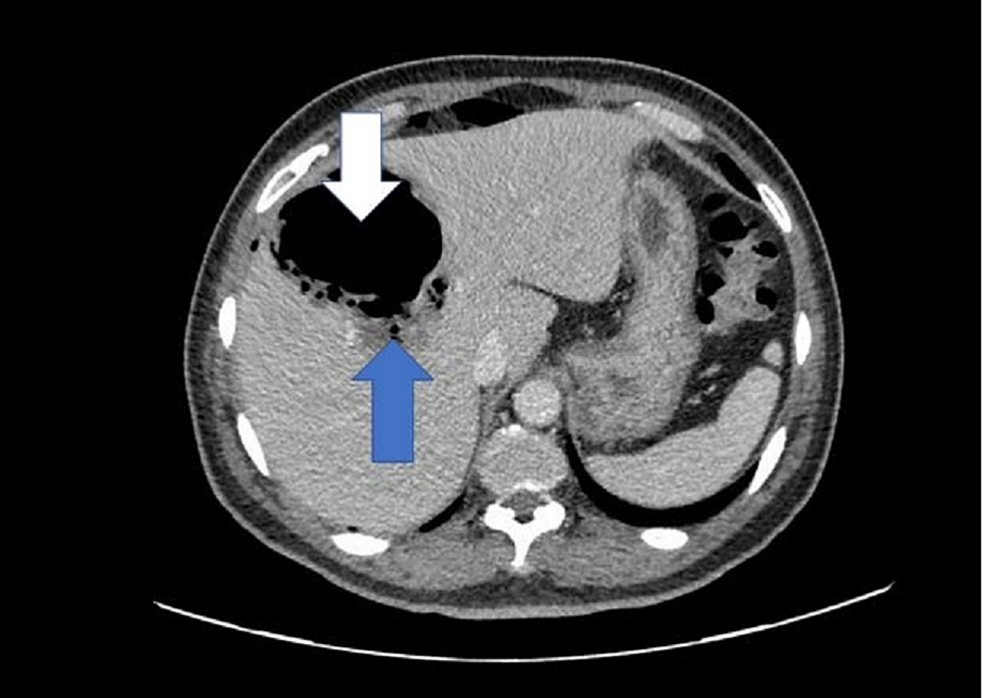
CT scan of the abdomen with IV contrast (axial view) IV: Intravenous CT scan image shows a large right sub-capsular hepatic lesion with an irregular outline, predominantly containing gas (white arrow) with minimal dependent fluid (blue arrow), suggestive of gas-forming hepatic abscess

Based on the clinical features and investigations, a provisional diagnosis of pneumoperitoneum with sepsis and uncontrolled diabetes was considered, with possible rupture of hollow viscus and peritonitis. A sample was collected for septic workup, and empirical antibiotics (piperacillin/tazobactam) and metronidazole were initiated. Hyperglycemia was managed with insulin and intravenous fluids. An urgent computerized tomography scan (CT scan) of the abdomen with contrast was performed, which showed a large right subcapsular hepatic lesion of 11.5 cm x 8.5 cm x 9 cm size with an irregular outline, predominantly containing gas with dependent fluid suggestive of gas-forming hepatic abscess. There was a possibility of rupture, with an associated pneumoperitoneum in the form of free air in the right subphrenic region along with the anterior and inferior surface of the liver (Figures 2-4).

.

**Figure 5:**
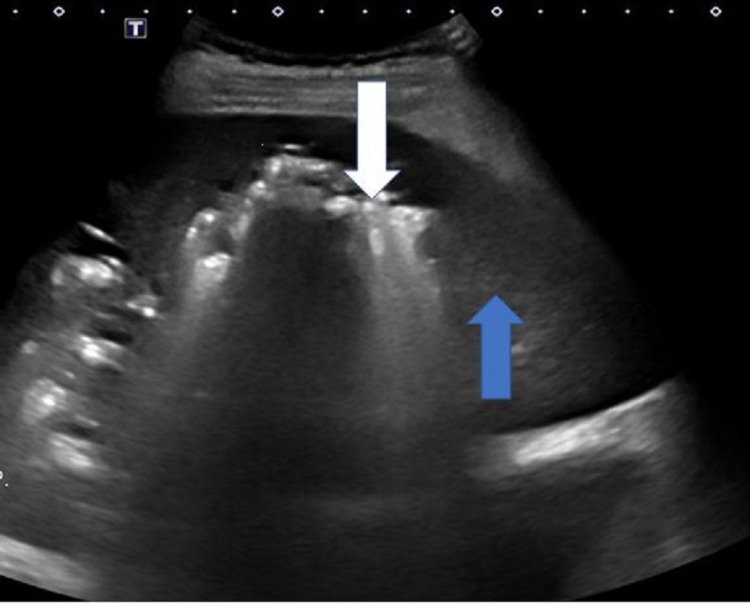
Gray-scale ultrasound of the liver The ultrasound image is showing a highly reflective bright shadow of the gas-containing abscess (white arrow) and normal adjacent liver parenchyma (blue arrow)

**Figure 6:**
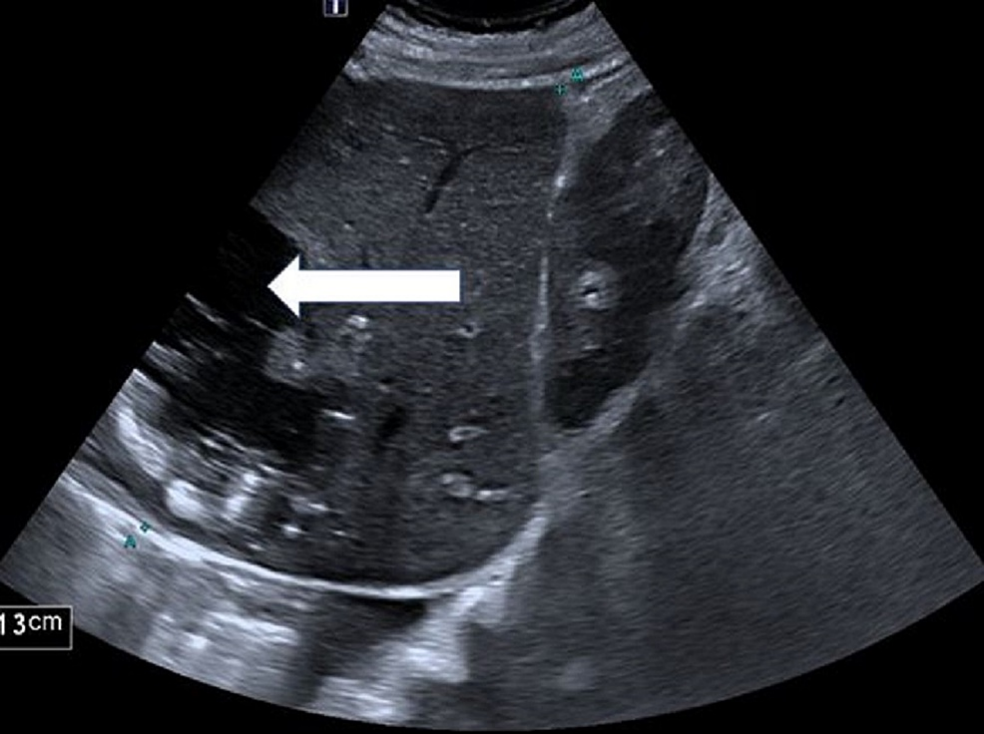
Ultrasound scan (post aspiration) The white arrow is showing a replacement of the gas with more fluid appearing as hypoechoic material

Under ultrasound guidance, aspiration of the abscess was done, and 125 ml of pus was drained (Figures 5, 6).

**Figure 7:**
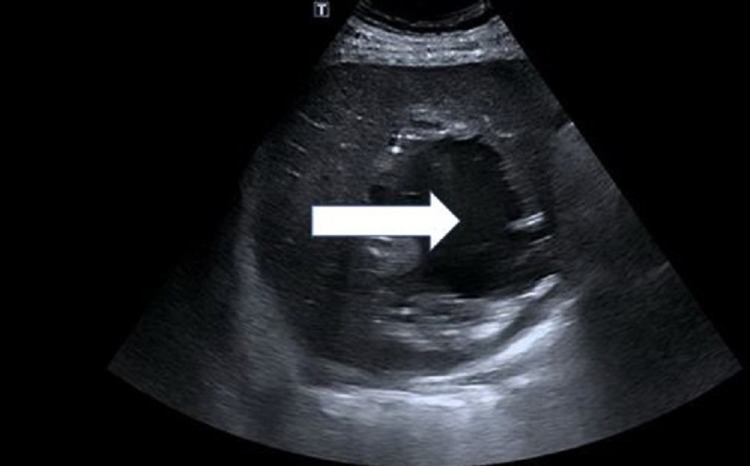
Repeat ultrasound of the abdomen The ultrasound image is showing an increase in the size of the gas-forming liver abscess (repeated after the fifth day of aspiration). Note the transformation of the contents of the abscess to liquid hypoechoic fluid (white arrow)

Blood and pus culture grew *Klebsiella pneumoniae*, and the antibiotic was changed to ampicillin/sulbactam based on the sensitivity result. The pain and laboratory markers started showing improvement following drainage and antibiotics. However, on the fifth day of admission, he again complained of pain over the right shoulder and right upper abdomen. Repeat ultrasound of the abdomen revealed re-demonstration of previously seen liver abscess with large volume, about 590 mL (Figure 7).

Repeat aspiration drained 30 mL pus, and an 8.5 Dawson Mullier drainage catheter (Cook Medical LLC, Bloomington, USA) was inserted into the abscess. 650 mL pus mixed with blood was drained over the next 24 hours. His clinical condition showed significant improvement with concomitant improvement in inflammatory markers. The antibiotic was de-escalated to intravenous ceftriaxone. Repeat ultrasound abdomen was performed (on day 30), which showed a small amount of residual organized phlegmon, unsuitable for aspiration. As the draining ceased, the catheter was removed, and the patient was discharged with a plan to continue antibiotic for a total of six weeks. On follow-up after four weeks, the patient was doing fine, and the repeat ultrasound was unremarkable.

## Discussion

Acute abdominal pain with gas under the diaphragm (pneumoperitoneum) is a medical emergency requiring urgent evaluation and management. In pneumoperitoneum, there is free air in the peritoneal cavity. It is diagnosed by an erect X-ray of the chest or abdomen. The most common cause is perforated hollow viscus (stomach, small or large bowel), constituting around 90% of the cases [1]. The other causes include intra-abdominal sepsis by gas-forming organisms, biliary-enteric fistula, post laparotomy/laparoscopic procedure, gall stone ileus, post scuba diving, and pneumatosis coli [2-4].

Rupture of liver abscess induced by a gas-forming organism is one of the rare causes of pneumoperitoneum. Gas forming pyogenic liver abscess (GFPLA), also known as emphysematous liver abscess, has the highest incidence in Southeast Asia, especially Taiwan [5]. It has an incidence ranging up to 30% of all pyogenic liver abscesses and has higher mortality than non-GFPLA (30.3% vs. 9 %) [6]. Reports show that the most common organism causing GFPLA is *Klebsiella pneumoniae*. In a study from Taiwan enrolling 22 cases of GFPLA, *K. pneumoniae* bacteremia was noted in 17 patients (77%) [7]. Other organisms causing GFPLA include *Escherichia coli* (*E. coli*), *Pseudomonas*, *Salmonella* spp, and *Clostridium* spp. *Salmonella* is a rare pathogen for GFPLA [8]. In the present case, *Klebsiella pneumoniae *was grown in blood and pus culture.

Gas forming liver abscess has been more common in diabetic patients than non-diabetic patients [9]. Moreover, diabetic patients had more complications than non-diabetics. Studies have shown that in patients with pyogenic liver abscess (PLA), the proportion of people with diabetes mellitus is 36.6-48.3% and in GFPLA 83.5-85.5% [6,9-12]. In the present case, the patient had diabetes for the last three years, which was poorly controlled (blood glucose: 43.1 mmol/L and HbA1c: 14.9%) owing to being non-compliant with the treatment. 

Gas associated with infection usually consists of nitrogen and carbon dioxide released via glucose fermentation by gas-forming bacteria. These bacteria derive their energy mainly from glucose by fermentation through various pathways, and mixed acid fermentation is the most preferred pathway [7]. The hyperglycemia in diabetes mellitus provides a favorable environment for the microorganisms to produce gas, as happened in the present case. Moreover, local tissue damage by the infection and the diabetic microangiopathy might slow the disposal of the catabolic end-products from the site, thus adding to the gas accumulation. A study involving analysis on the composition of gas aspirated from GFPLA patients due to *Klebsiella pneumoniae* found that nitrogen was the predominant gas detected in all the samples. The other gases were carbon dioxide, hydrogen, and oxygen [7]. 

GFPLA diagnosis is usually made by radiological images demonstrating gas in the liver parenchyma. Plain (erect) abdominal radiographs show abnormal gas patterns, air-fluid levels and mottled gas patterns being the most common findings, but the plain radiographs are less reliable. Gas formation in the liver parenchyma is noted in only up to 36% of patients with GFPLA on plain radiograph films [7]. Free air under the diaphragm denotes rupture of the abscess. Ultrasonography or CT scan are reliable diagnostic imaging modalities for GFPLA. A detection rate of 100% has been reported with either US or CT scan [7].

In our patient, the plain film revealed right subdiaphragmatic free air suggesting pneumoperitoneum with heterogeneous liver shade and gas-fluid level. CT scan confirmed features suggesting gas-forming hepatic abscess and pneumoperitoneum.

Treatment of GFPLA includes appropriate antibiotic cover, adequate drainage to improve the tissue perfusion to facilitate gas transport, and good control of blood sugar. GFPLA has been traditionally managed by surgical exploration and drainage. The last few decades witnessed a marked shift from the traditional surgical exploration and drainage practice to image-guided percutaneous transhepatic drainage. 

A study examining 22 cases of emphysematous liver abscess reported percutaneous transhepatic abscess drainage in 19 patients and surgery in one case [7]. Drainage is particularly recommended in cases of large abscesses exceeding 5 cm in diameter. In cases of rupture and peritonitis, surgery should be considered [5,8]. Although percutaneous drainage has replaced surgery as the primary treatment of liver abscesses, the surgery still has its essential role under some conditions. A surgical drainage is an option when the underlying disease demands primary surgical management. Inadequate response to catheter drainage or if the abscess has viscous contents which could not be aspirated are other indications for surgical drainage [13].

In our patient, though it was a ruptured GFPLA, instead of doing surgical drainage, the surgical team and the interventional radiologist opted for percutaneous drainage under ultrasound guidance, followed by deployment of catheter drain, and the patient improved without requiring open surgery.

## Conclusions

Most of the cases of acute abdominal pain with pneumoperitoneum are attributable to perforated hollow viscus. However, other possibilities like rupture of liver abscess (by the gas-forming organism) also need to be considered. Further imaging like a CT scan might help diagnose and obviate the need for laparotomy in some cases. Our patient was successfully managed with ultrasound-guided percutaneous aspiration, drain insertion, and antibiotics, along with supportive measures. 
